# Assessing bystander first aid: development and validation of a First Aid Quality Assessment (FAQA) tool

**DOI:** 10.1186/s12873-023-00811-z

**Published:** 2023-04-04

**Authors:** Siri Idland, Jo Kramer-Johansen, Håkon Kvåle Bakke, Magnus Hjortdahl

**Affiliations:** 1 Institute of Nursing and Health Promotion, Faculty of Health Science, Bachelor Program in Paramedic Science, Oslo Metropolitan University, Oslo, Norway; 2 Division of Prehospital Services, Oslo University Hospital, Oslo, Norway; 3 Institute of Clinical Medicine, University of Oslo, Oslo, Norway; 4 Department of Anaesthesia and Critical Care, University Hospital of North Norway, Tromsø, Norway; 5 Department of Health and Care Sciences, Faculty of Health Science, UiT, The Arctic University of Norway, Tromsø, Norway

**Keywords:** First aid, Injury, Trauma, Bystander, Validate

## Abstract

**Background:**

Injuries are one of the leading causes of death worldwide. Bystanders at the scene can perform first aid measures before the arrival of health services. The quality of first aid measures likely affects patient outcome. However, scientific evidence on its effect on patient outcome is limited. To properly assess bystander first aid quality, measure effect, and facilitate improvement, validated assessment tools are needed.

The purpose of this study was to develop and validate a First Aid Quality Assessment (FAQA) tool. The FAQA tool focuses on first aid measures for injured patients based on the ABC-principle, as assessed by ambulance personnel arriving on scene.

**Methods:**

In phase 1, we drafted an initial version of the FAQA tool for assessment of airway management, control of external bleeding, recovery position and hypothermia prevention. A group of ambulance personnel aided presentation and wording of the tool. In phase 2 we made eight virtual reality (VR) films, each presenting an injury scenario where bystander performed first aid. In phase 3, an expert group discussed until consensus on how the FAQA tool should rate each scenario. Followingly, 19 respondents, all ambulance personnel, rated the eight films with the FAQA tool. We assessed concurrent validity and inter-rater agreement by visual inspection and Kendall’s coefficient of concordance.

**Results:**

FAQA-scores by the expert group concurred with ± 1 of the median of the respondents on all first aid measures for all eight films except one case, where a deviation of 2 was seen. The inter-rater agreement was “very good” for three first aid measures, “good” for one, and “moderate” for the scoring of overall quality on first aid measures.

**Conclusion:**

Our findings show that it is feasible and acceptable for ambulance personnel to collect information on bystander first aid with the FAQA tool and will be of importance for future research on bystander first aid for injured patients.

**Supplementary Information:**

The online version contains supplementary material available at 10.1186/s12873-023-00811-z.

## Background

Injuries is one of the leading causes of death worldwide [1]. Bystanders are often first at scene, and present at the arrival of the ambulance services [2]. Until the arrival of professional emergency medical service, bystanders are often advised to perform first aid for the injured person, starting with checking for a response, normal breathing and external bleedings, [3] and with life-saving measures to be performed according to the same systematic approach. Some scientific knowledge on effects of bystander first aid on patient outcome exists, but mainly on out-of-hospital cardiac arrest and cardiopulmonary resuscitation (CPR) [1].

There is little knowledge about the potential impact of bystander first aid for injured patients, and scarce research on the correlation between quality of first aid and patient outcome [4]. One way to collect data on bystander first aid, is to engage ambulance personnel to evaluate bystander’s actions a critically ill or injured patient. In three published studies, bystander first aid on injuries have been evaluated by non-validated tools [2, 5, 6]. Assessment of bystander first aid involves elements of subjectivity by the scorer. The development and validation of a tool for ambulance personnel to assess bystander’s first aid action will both determine whether previous studies can be trusted, and facilitate future studies.

We wished to develop and validate a bystander first aid assessment tool aimed for ambulance personnel to assess bystander first aid for injured patients, the First Aid Quality Assessment (FAQA) tool. We focused on injured patients and emphasized feasibility for quick scoring to avoid treatment delays. Based on first aid guidelines, we limited our assessment to airway management, stopping external bleeding, recovery position to maintain open airway, and prevention of hypothermia. The purpose of this study was to develop and validate the FAQA tool, and thus provide a validated tool for future research on bystander first aid for injured patients.

## Methods

### Phase 1: development of the FAQA tool

The tool we have developed is based on previous work by Bakke et al. [2, 7]. In phase 1 of the development and validation process, the project group had several discussions about how the tool should be developed further, based on experiences from the previous studies where the original questionnaire was used [2, 7]. As bystander first aid guidelines recommend to begin first aid with the ABCDE-principle [3], we chose to focus on airway management, bleeding control, recovery position and hypothermia prevention. The tool is accessed by a web-browser on a smartphone or a tablet, which transfers the data through a safe connection directly to an approved server for storage on sensitive data.

For face validity, we gathered a group of five ambulance personnel to assess the first draft of the FAQA tool before it was considered as finalized. They gave their feedback through group discussion resulting in changes in the order of the first aid measures on the tool, and improved wording and sentence building.

**Fig. 1:**
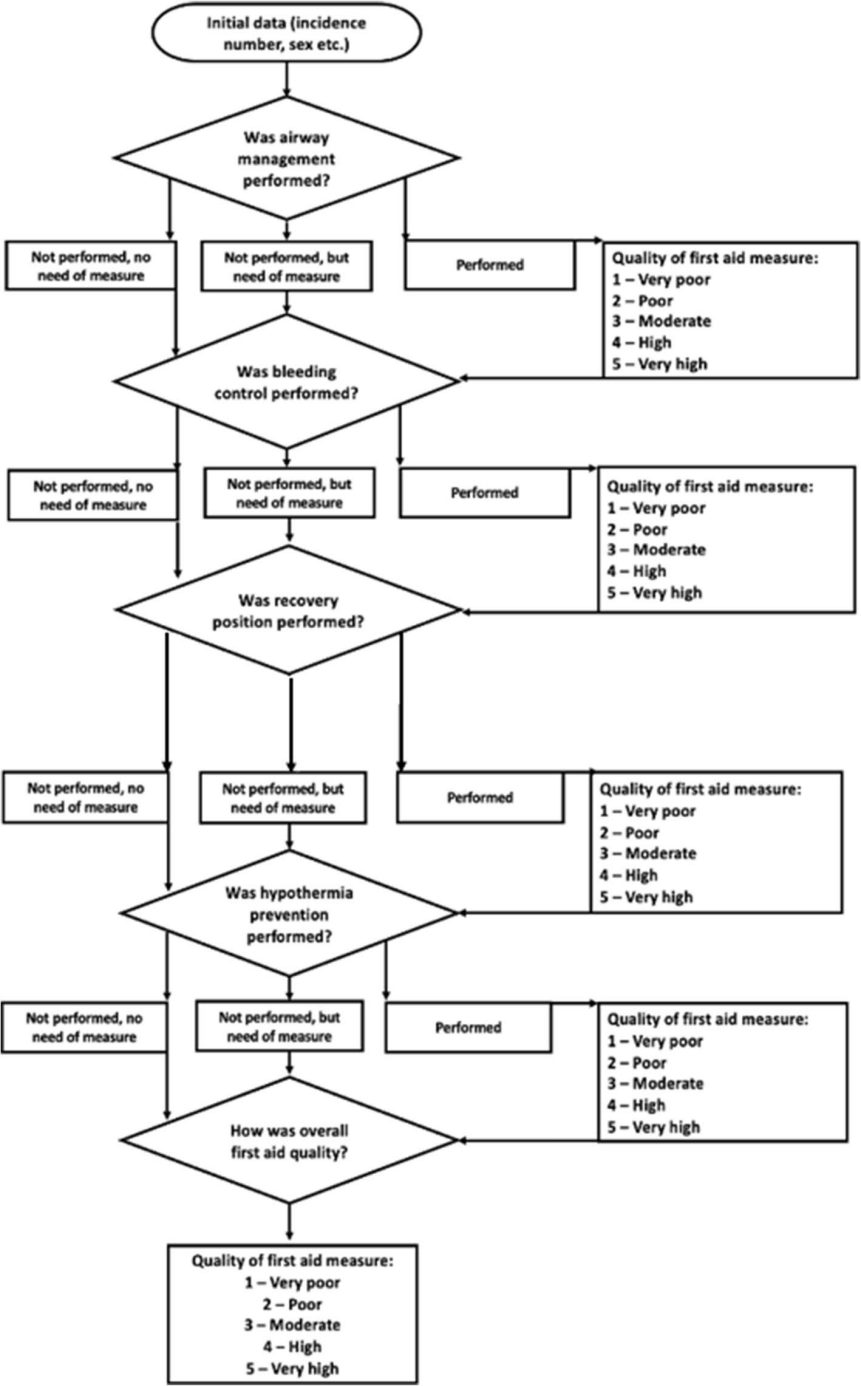
Flow chart illustrating the items in the FAQA-tool

The tool is accessed on a tablet or a smartphone (see Additional file 1), and total number of items in the tool is 10 to 14, depending on the respondents’ answer. For information on first aid, ambulance personnel will register the following information on each of the four first aid measures mentioned below, in addition to overall quality on all first aid measures performed:

- Whether bystanders had performed or should have performed each of the four measures: airway management, bleeding control, recovery position and hypothermia prevention. This question has three options: 1) performed, 2) not performed, the patient was not in need of the measure, 3) not performed, but the patient was in need of this measure.
- If performed, quality of performance on a Likert scale from 1 (very poor) to 5 (optimal) for this first aid measure (see Fig. 1).

### Phase 2: production of VR films

To validate the tool, we produced eight short films with BASIC virtual reality (VR) technology. All films created for the validation process have different stories with focus on first aid performed by bystander with various quality, just as ambulance personnel arrives at the scene (described in Table 1). The project group created the films, and the directing and filming were executed by a professional company specialized in developing VR-training for groups such as in the military and in health care.

**Table 1:** Overview of the eight films developed for the validation process

	Background	State of patient	Situation at the arrival of ambulance	Bystander first aid measures at the arrival of ambulance
Film 1	Female driver in traffic accident. Two bystanders present	Unconscious. Blocked airways	Driver brought out of the car, lying on the ground	Bystander 1 have lifted driver’s jaw, opened mouth
Film 2	Male driver in traffic accident. Several bystanders present	Unconscious. Blocked airways. Modest bleeding from arm	Chaotic situation. Man sitting in driver seat with blocked airways (snoring)	One bystander holds a cloth loosely to the modest bleed
Film 3	Construction worker with severe cut from disk saw. Several colleagues present	Awake, has a severe cut on forearm	Sitting in upright position leaning backwards at wall Two colleagues handling the bleed	Colleague 1 lifting the forearm, the other having bound a cloth tightly over wound, pressing down Blanket lain over man’s shoulders
Film 4	Man cut on log-splitter in private garden. 2 neighbors present	Awake. Severe cut in hand	Sitting in upright position on the ground, the neighbors standing a few meters away	The man has been given a cloth, severe bleed from wound
Film 5	Man fallen from house roof (approx 6 m). Several bystanders present	Unconscious, breathing	Man lying on the ground. Two bystanders taking care of the man	Patient in recovery position. A jacket has been lain under him, a blanket on top of him
Film 6	Old man, in-door fall. Wife and son present	Unconscious, breathing	Man lying on the floor. Wife and son present in the same room	Lying in supine position. No measures performed
Film 7	Car driven off the road. Driver sole person in the car. Several bystanders present	Unconscious, breathing	Brought out of the car	Jacket lain under him as well as on top of him. Dressed in a warm hat
Film 8	Woman fallen off bike, been riding her bike in the woods. Several bystanders present	Unconscious, breathing	Lying beside her bike. Thin clothing	Patient in recovery position

### Phase 3: validation of the FAQA tool

In phase 3, an expert group watched the filmed first aid scenarios and used the FAQA tool to score each film. If scores differed, the group discussed until consensus was found. The expert consensus was considered the gold standard for how each scenario should be rated using the assessment tool. The expert group consisted of four members with various backgrounds (Table 2).

**Table 2:** Composition of expert group: clinical specialty and years of clinical experience

	Profession	Clinical specialty/field of experience	Clinical experience (years)
Expert 1	General practitioner	Ambulance service, out-of-hours primary emergency clinic	17
Expert 2	Anaesthesiologist	Department of anaesthesia, ambulance service	24
Expert 3	Paramedic	Ambulance service	24
Expert 4	Nurse	Emergency department	10

Followingly, we recruited 19 voluntary areas ambulance personnel as respondents. They were all trained as emergency medical technicians (EMTs). Many of them were also trained nurses or national paramedics. Their amount of experience in the ambulance service varied from 6 to 20 years. All the volunteers were also training officers in their area. They watched the eight films with VR glasses and completed the assessment tool after each film.

When validating the FAQA tool, the main components of validity and reliability relevant were inter-rater agreement and concurrent validity. For assessment of the inter-rater agreement, the extent/degree of agreement among the respondents was investigated [8]. We tested the inter-rater agreement with Kendall’s coefficient of concordance, using the interpretation of Landis and Koch when presenting the results [9]. Concurrent validity is when two assessments agree, or a new measure is compared favorably with one that is considered valid [10]. To measure this, we compared the degree of agreement between the participating respondents with the expert group. This was done by visual inspection. A priori we decided that an agreement of ± 1 on the scale of quality (1–5) would be considered adequate for this measure.

For analyzation and presentation of the results, 0 represents “not performed, but the patient was in need of this measure” -1 represents “not performed, the patient was not in need of the measure”.

## Results

We developed a tool for assessing bystander first aid measures at arrival of ambulance personnel with ten to fourteen items. Nineteen ambulance personnel viewed eight filmed scenarios where bystanders had performed first aid, and then used the electronic FAQA tool to assess bystander first aid after each film. All respondents completed the tool for all films except for film 3, where 18 responses were registered. In total, the FAQA tool was completed 151 times by the respondents.

### Inter-rater agreement

The agreement of the respondents regarding whether first aid measures were performed varied. Inter-rater reliability among respondents was computed for all four first aid measures in addition to overall quality on first aid measures. Highest agreement between raters was found for bleeding control measures and recovery position, whereas the lowest agreement between raters was found for airway management measures and overall quality (Table 3).

**Table 3:** Inter-rater agreement shown by Kendall’s coefficient of concordance

	Kendall’s W	Concordance
Airway management	0,660	Good agreement (0,61–0,80)
Bleeding control	0,975	Very good agreement (> 0,80)
Recovery position	0,838	Very good agreement (> 0,80)
Hypothermia prevention	0,801	Very good agreement (> 0,80)
Overall quality of first aid measures	0,570	Moderate agreement (0,41- 0,60)

Kendall’s W ranges from 0 to 1, where 1 represent identical ratings [9]

### Concurrent validity

**Fig. 2:**
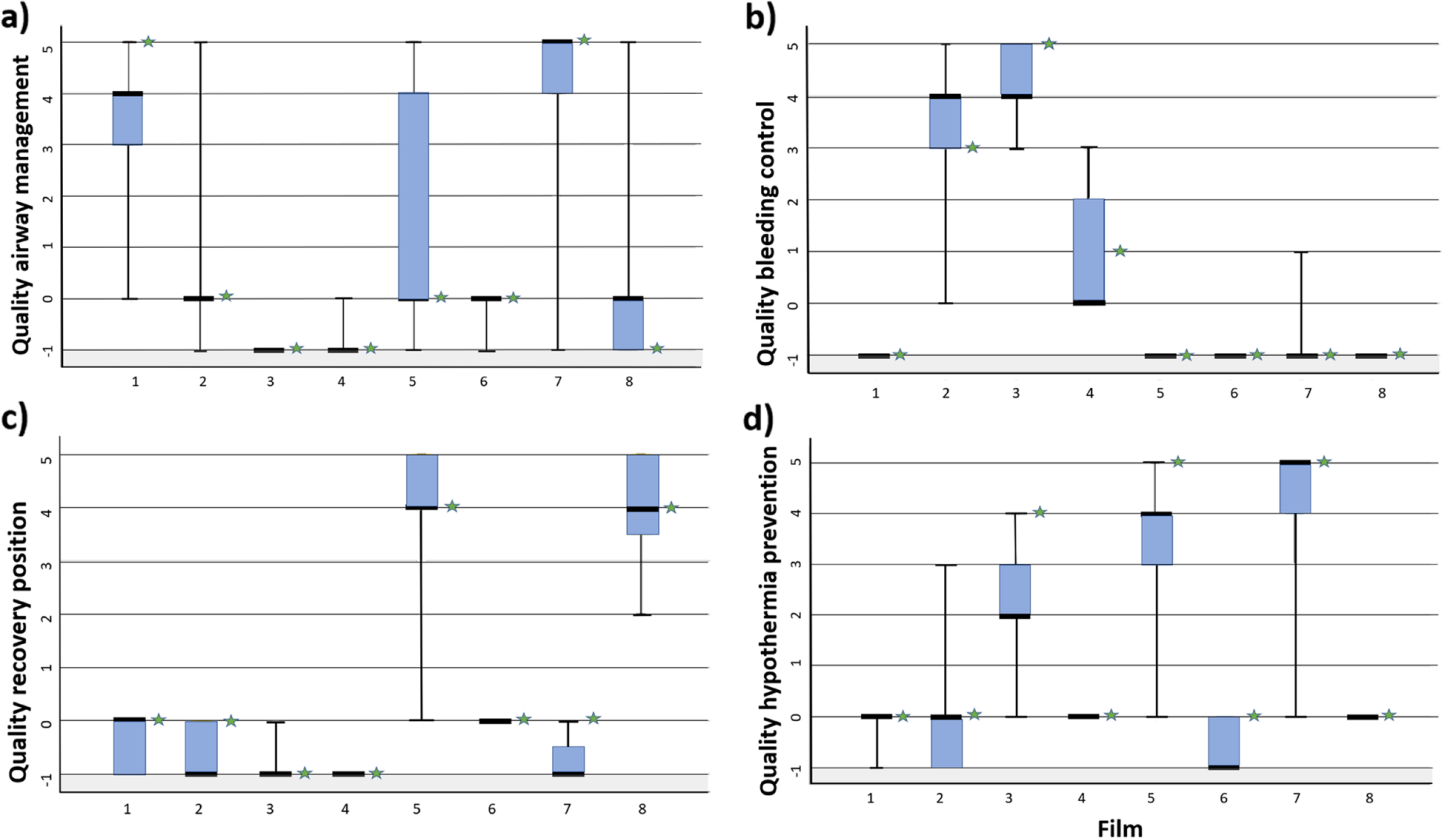
**a**, **b**, **c**, **d** Box-plot of respondents’ assessment of bystander’s performance of first aid measures, as well as opinion of the expert group, for each film (1–8) for each of the four interventions **a** airway management, **b** bleeding control, **c** recovery position, and **d** hypothermia prevention). The whiskers in the box-plots represent the complete range for each film, outliers included. -1 – 5 represents whether the four first aid measure were performed and quality of performance. -1 = not performed, the patient was not in need of the measure. 0 = not performed, but the patient was in need of the measure. 1 = very poor quality, 2 = poor quality, 3 = moderate quality, 4 = high quality, 5 = very high quality.  : expert opinion

For most of the films, the expert opinion concurred with the median ± 1 of the respondents’ answers on all first aid measures. The largest deviation seen was a deviation of 2. These findings are presented in Fig. 2.

**Fig. 3:**
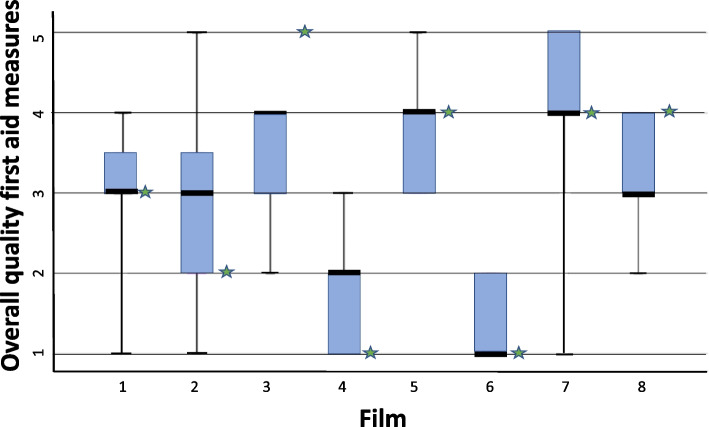
Box-plot of respondents’ assessment of bystander’s overall quality of first aid measures, as well as opinion of the expert group, for each film (1–8). The whiskers in the box-plots represent the complete range for each film, outliers included. 1 – 5 represents rating of overall quality on first aid measures performed. 1 = very poor quality, 2 = poor quality, 3 = moderate, 4 = high quality, 5 = very high quality.  : expert opinion

For rating of overall quality of first aid measures, the expert opinion deviated with 1 as a maximum from the median of the respondents, see Fig. 3.

## Discussion

The aim of this study was to develop and validate the FAQA tool to assess bystander first aid on injured patients, rated by ambulance personnel. No such validated tools exist, and our development and validation of this tool will hence be of importance for further research on bystander first aid and patient outcome. Our findings show that the inter-rater reliability of first aid measure performance varied from moderate to very good agreement. The concurrent validity, i.e., agreement between respondents and expert group, diverged with a maximum of two when comparing the expert group opinion and the median of the respondents.

### Development of the FAQA tool

The International Federation of the Red Cross and Red Crescent Societies (IFRC) recommends bystanders to use a systematic approach when assessing injured people, based on the ABCDE-principle [3]. We included the four first aid measures in the tool because we believe them to be the most commonly performed measures, as well as those with highest potential impact in initial bystander first aid performance. Cardiopulmonary resuscitation (CPR) was considered but was not included in the FAQA tool. CPR would be relevant for a minority of injured patients and assessment of CPR quality is arguably more important for patients with a non-traumatic cause of cardiac arrest. We worried that adding CPR as a first aid measure in our FAQA-tool might bring some confusion to ambulance personnel completing the questionnaire in whether cardiac arrest cases with a cardiac cause should be included. There are several first aid measures, for instance burn injury treatment and immobilization of fractures, that could be included in a tool like the FAQA tool. Our aim was to develop a tool that is relevant (focusing on life saving firs aid procedures) and feasible (easy to understand and to perform, and not too extensive). This led us to select airway management, control of external bleeding, recovery position and hypothermia prevention.

Previous studies have evaluated bystander first aid on injured patients with the aid of ambulance personnel, but the tools used have not been validated. However, other tools used in scientific work of emergency care has been validated with approaches similar to ours. As an example, tools to measure teamwork and non-technical skills during resuscitation has been developed, based on an existing evidence base [11, 12]. Some research implies that bystander first aid for injured patients may decrease mortality [13, 14]. The effects of bystander first aid for injured patients lack scientific evidence [1, 2, 14], and research on this field requires validated tools as a means to collect reliable data. Effects on bystander CPR before the arrival of professional health services are well known [1], and we believe that similar effects of first aid measures on injured patients deserve scientific attention.

### Reliability and validity

Several interpretations of Kendall’s W have been presented, one of them to extend the interpretation of kappa as described by Landis and Koch [9] to Kendall’s W. Using this interpretation, strength of agreement of airway management was good. This may be due to several factors. We believe that assessing airway management as an isolated measure, excluding recovery position, might bring disagreement among raters. This may be one of the reasons why the inter-rater reliability of airway management is lower than for the other first aid measures.

Visual inspection revealed that for almost all first aid measures in the films, the disagreement between the median of the respondents’ answers and expert group did not exceed one. There was a single case with a difference of two, (hypothermia prevention measures in film three). In a clinical setting, we suggest that this is satisfactory. There will always be some subjectivity when scoring opinions, which is why we considered a disagreement of one as acceptable for this setting.

Our findings reveal somewhat lower agreement among the respondents on overall quality of first aid measures, as well as between the expert group and the respondents. We believe that rating overall quality of first aid measures naturally diverges more than the rating of one specific measure, as the question of overall quality was an open question without specific examples.

For some first aid measures, there was disagreement among the respondents as well as between the respondents and the expert group whether a first aid measure was performed with very poor quality or whether the first aid measure was performed at all. We argue that the gliding transition from not performing to performing with very low quality is present in real scenarios as well, and that the disagreement we found is to be expected.

### Strengths and limitations

This first aid quality assessment tool has focused on the immediate lifesaving measures of first aid in trauma, airways and circulation i.e. the ABC of the ABCDE. With “good” to “very good” inter-rater agreement on complex measures such as airway management and control of external bleeding, the tool should be possible to expand to the concrete measures of the D and E, such as the cooling of burns or stabilization of fractures. With such an expansion of the tool, validation studies of the tool should be repeated.

The respondents in this study were all ambulance personnel with different backgrounds. All of them also have a training officer responsibility in their health trust. Even though they all have a training officer responsibility, we argue that the results can be generalized to other personnel with similar training because of their varied educational background and number of years in work-experience as ambulance personnel. None has less work-experience than five years, which may limit the generalizability of the results to ambulance personnel with few years of experience.

When validating tools for scientific purposes like ours, ideally convergent and discriminant validity should be computed. In our study this is difficult to execute because to our knowledge, no similar tools to the FAQA tool to compare with exists. In addition, the number of respondents pose a limitation to the study as a larger number would give more power to the computed statistical analysis. However, we believe that the number of respondents in this study is satisfactory as the group is somewhat homogenous being all ambulance personnel, the total number of times the tool was completed is 151, and due to the novelty of the tool.

The films used are also a possible limitation of this study. However, we believe the development of these films as the best option to validate the tool, as no other fit option for reproducible settings for scoring with the tool exists. To illustrate the situations as close to real life as possible, we chose to use VR technology, and the films were shot from the ambulance personnel’s view. With this method, we believe the participants’ feelings of real-life situations were enhanced. In addition, we believe VR contributed to the respondents’ participation in the project. All scenarios were written and edited in cooperation by several members in the project group before filming.

We believe that the validation of the FAQA-tool to some extent can contribute to control for subjectivity. Nonetheless, the problem of subjectivity in this type of scoring tool cannot be avoided and will still have to be addressed in studies using the tool. We view the tool as a first step of a validated tool to assess bystander first aid, and that it may be improved further.

## Conclusion

In this study we developed a First Aid Quality Assessment tool for assessing bystander first aid on injured patients. The validation showed inter-rater agreement above good for all concrete assessment items, and moderate for assessment of overall quality. The correlation between how the bystander performs first aid for injured patients and patient outcome is not known. To our knowledge, this is the only assessment tool where ambulance personnel evaluate bystander first aid on injured patients which has been validated. We believe that the opportunity to use the FAQA tool in future studies may contribute to important knowledge on this subject.

## Supplementary Information

**Additional file 1.** The First Aid Quality Assessment (FAQA) tool for evaluation of bystander first aid on injured patients by ambulance personnel.

## Data Availability

The datasets used and/or analyzed during the current study are available from the corresponding author on reasonable request.

## Abbreviations

CPR: Cardiopulmonary resuscitation
EMT: Emergency medical technician
FAQA: First Aid Quality Assessment
IFRC: The International Foundation of the Red Cross and Red Crescent Societies
VR: Virtual technology
