# Supplementary material for: Assessing bystander first aid: development and validation of a First Aid Quality Assessment (FAQA) tool
Source: BMC Emerg Med. 2023 Apr 4;23:39. doi: 10.1186/s12873-023-00811-z (PMC10071655; doi:10.1186/s12873-023-00811-z)
Supplement: Supplementary file 1 — Additional file 1. The First Aid Quality Assessment (FAQA) tool for evaluation of bystander first aid on injured patients by ambulance personnel. [file 12873_2023_811_MOESM1_ESM.pdf]

Additional file 1: The First Aid Quality Assessment (FAQA) tool for evaluation of bystander first aid by ambulance personnel

The First Aid Quality Assessment (FAQA) tool for evaluation of bystander first aid by ambulance personnel

Page 1

Mandatory fields are marked with a star *

Ambulance personnel fill out the questionnaire for all cases where:

- the patient has an **injury**
- there is more than one person present in addition to the patient (a potential first aid performer)
- one or more of the following first aid measures have been conducted or should have been conducted before the arrival of the ambulance personnel:

-airway management

- external bleeding control

-recovery position

-hypothermia prevention

Page break

Page 2

Mandatory fields are marked with a star *

Incident number *

The incident number of the mission

Sex of patient

- Male
- Female

Age of patient

0 10 20 30 40 50 60 70 80 90

Verdi

Estimated age or exact age

- Exact
- Estimated

Page break

Page 3

Mandatory fields are marked with a star *

Who is the bystander(s) performing first aid?

Register whether the bystander performing first aid is from the police, fire brigade or a civilian first aid responder who has been called to the incident

- Police or fire brigade
- Civilian first aid responder (not police or fire brigade)
- Lay persons

Airway management: Did bystander attempt airway management on the patient?

Examples: Chin lift, jaw thrust

- Performed
- Not performed, the patient was NOT in need of this measure
- Not performed, but the patient WAS in need of this measure

Quality of airway management

This element is only shown when the option "Performed" is selected in the question "Airway management: Did bystander attempt airway management on the patient?"

How would you evaluate the quality of bystander's airway management of the patient?

- Very poor
- Poor
- Moderate
- High
- Very high

External bleeding control: Did bystander attempt bleeding control on the patient?

Examples: Direct pressure, lifting of extremity, stop the bleed with e.g. cloth or with torniquet

- Performed
- Not performed, the patient was NOT in need of this measure
- Not performed, but the patient WAS in need of this measure

Quality of bleeding control

This element is only shown when the option "Performed" is selected in the question "External bleeding control: Did bystander attempt bleeding control on the patient?"

How would you evaluate the quality of bystander's attempt on bleeding control?

- Very poor
- Poor
- Moderate
- High
- Very high

Recovery position: Did bystander attempt to put the patient in recovery position?

- Performed
- Not performed, the patient was NOT in need of this measure
- Not performed, but the patient WAS in need of this measure

Quality of recovery position

This element is only shown when the option "Performed" is selected in the question "Recovery position: Did bystander attempt to put the patient in recovery position?"

How would you evaluate the quality of bystander's attempt on putting the patient in recovery position?

- Very poor
- Poor
- Moderate
- High
- Very high

Hypothermia prevention: Did bystander attempt to perform measures o[illegible] the patient to prevent hypothermia?

Examples: isolating layers between the patient and the ground, shield the patient by bringing him/her inside e.g. a house or a car, cover the patient with e.g. wind canvas/sleeping bag/blanket, removal of patient's moist/wet clothing.

- Performed
- Not performed, the patient was NOT in need of this measure
- Not performed, but the patient WAS in need of this measure

Quality of measures for hypothermia prevention

This element is only shown when the option "Performed" is selected in the question "Hypothermia prevention: Did bystander attempt to perform measures on the patient to prevent hypothermia?"

How would you evaluate the quality of bystander's attempt to prevent the patient from hypothermia?

- Very poor
- Poor
- Moderate
- High
- Very high

Overall quality of first aid measures

How would you evaluate the overall quality of bystander's performance of lifesaving first aid measures on the patient?

- Very poor
- Poor
- Moderate
- High
- Very high

Page break
